# Correlation between Hyperkalemia and the Duration of Several Hospitalizations in Patients with Chronic Kidney Disease

**DOI:** 10.3390/jcm11010244

**Published:** 2022-01-04

**Authors:** Vincenzo Calabrese, Valeria Cernaro, Valeria Battaglia, Guido Gembillo, Elisa Longhitano, Rossella Siligato, Giovanna Sposito, Guido Ferlazzo, Domenico Santoro

**Affiliations:** 1 Unit of Nephrology and Dialysis, Department of Clinical and Experimental Medicine, University of Messina, 98125 Messina, Italy; v.calabrese@outlook.it (V.C.); vcernaro@unime.it (V.C.); vale-battaglia@libero.it (V.B.); guidogembillo@live.it (G.G.); elisa.longhitano@libero.it (E.L.); rossellasiligato@gmail.it (R.S.); 2 Unit of Clinical Pathology, Department of Human Pathology of Adults and Developmental Age, University of Messina, 98125 Messina, Italy; giovanna.sposito@unime.it (G.S.); guido.ferlazzo@unime.it (G.F.)

**Keywords:** chronic kidney disease, hospitalization, hyperkalemia, serum potassium levels, renin–angiotensin–aldosterone system inhibitors

## Abstract

(1) Background: This observational study aimed to verify the association between serum potassium levels and hospitalization days in patients with chronic kidney disease in a follow up of nine months. (2) Methods: Patients with chronic kidney disease were divided into group A (180 patients, potassium ≤ 5.1 mEq/L) and B (90 patients, potassium > 5.1 mEq/L). Student’s *t*-test, Mann–Whitney test, Pearson’s Chi-Square test, Pearson/Spearman’s correlation test and linear regression test were performed in the entire sample and in stage-G4/5 subsample. (3) Results: Groups A and B differed for estimated glomerular filtration rate (eGFR) (34.89 (IQR, 16.24–57.98) vs. 19.8 (IQR, 10.50–32.50) mL/min/1.73 m^2^; *p* < 0.0001), hemoglobin (11.64 ± 2.20 vs. 10.97 ± 2.19 g/dL, *p* = 0.048), sum of hospitalization days (8 (IQR, 6–10) vs. 11 (IQR, 7–15) days; *p* < 0.0001) and use of angiotensin II receptor blockers (40.2% vs. 53.3%; *p* = 0.010). Considering patients with eGFR 6–30 mL/min/1.73 m^2^, differences in the sum of hospitalization days were confirmed. Multivariable regression analysis showed that hyperkalemia is an independent risk factor of increased hospital length. In stage G4-G5, regression analysis showed that hyperkalemia is the only independent risk factor (β = 2.93, 95% confidence interval, 0.077–5.794, *p* = 0.044). (4) Conclusions: We observed significantly greater odds of increased length of hospital stay among patients with higher potassium, mostly in stages G4–G5 chronic kidney disease.

## 1. Introduction

Potassium is the most abundant inorganic ion within cells; 98% of this cation (K+) is found in the intracellular compartment (about 150 mEq/L) and 2% is found in the extracellular compartment (about 3–5 mEq/L) [1]. Potassium plays a key role in the maintenance of body homeostasis, and it is involved in many physiological functions, including regulation of electrolytes and acid-base balance and neuromuscular conduction. Na+/K+-ATPases help in maintaining this equilibrium [2]. Variations in potassium concentrations may alter the membrane potential and impair the central and peripheral nervous system, as well as neuromuscular and cardiac functions [3].

Hyperkalemia, defined as a concentration of serum potassium > 5.1 mEq/L, can be due to (1) impaired excretion of potassium in urine owing to renal failure; (2) renin–angiotensin–aldosterone system inhibitors (RAASIs); drugs interfering with aldosterone metabolism and renin release (heparin or ketoconazole and nonsteroidal anti-inflammatory drugs (NSAIDs) or cyclosporine) and with ion-channel function (β-blockers and digoxin in relation to the Na-K pump; amiloride, trimethoprim and pentamidine in relation to the Na channel); (3) shift from the intracellular compartment after intravenous infusion of glucose; (4) tumor lysis syndrome; (5) increased potassium intake, especially in patients with renal impairment [1,4,5,6].

However, according to recent studies, hyperkalemia is a major cause of impaired renal excretion [7,8,9,10]. The incidence of hyperkalemia increased from 2% to 42% if the estimated glomerular filtration rate (eGFR) decreased from 60 mL/min/1.73 m^2^ to 20 mL/min/1.73 m^2^ [11,12]. Moreover, age, male gender, heart failure (HF) with reduced ejection fraction and cancer are risk factors associated with hyperkalemia [6,13,14,15].

The changes in electrolytes due to fluctuations in potassium levels limit the use of RAASIs, which are the therapeutic strategies currently used to slow down progression of chronic kidney disease (CKD). Furthermore, hyperkalemia is a risk factor for mortality both independently and in association with age or RAASI use, and it may result in lethal arrhythmias [16,17,18].

The aim of the present study is to verify the association between serum potassium levels and the duration of several hospitalizations in patients with CKD admitted to the Unit of Nephrology and Dialysis of the University Hospital of Messina (Italy), also taking into account home therapies and comorbidities.

## 2. Materials and Methods

### 2.1. Population and Study Design

This prospective observational cohort study with retrospective enrollment was carried out from 1 April 2018 to 31 December 2018 at the Unit of Nephrology and Dialysis of the University Hospital of Messina (Italy) (Ethics Committee approval protocol: 11–21). We considered patients with first admission in this period, with a follow-up of nine months. Upon admission, patients sign an informed consent form regarding the use of clinical data for scientific purposes. All patients enrolled in the present study provided written consent. Data were retrieved from clinical records available at our ward. We included all admitted patients with a diagnosis of CKD at stages G1-G5 or A1-A3 or with other anatomic or urine sediment abnormalities according to the KDIGO 2012 Clinical Practice Guideline for the Evaluation and Management of Chronic Kidney Disease [19]. The exclusion criteria were death on the first day; transfer from or to other wards; discharge by voluntary choice; dialysis treatment, age <18 years; and admission to our ward in the last 12 months. Six hundred twenty-four adult patients were admitted to our ward. Among them, 270 patients met the inclusion criteria, 4 died in the first day of hospitalization, 72 patients needed of hemodialysis and 174 were transferred or discharged by voluntary choice. All available data were used in this study.

### 2.2. Study Procedures

The population was divided into two groups according to serum potassium levels: group A (potassium ≤ 5.1 mEq/L; *n* = 180 patients) and group B (potassium > 5.1 mEq/L; *n* = 90 patients). This limit of serum potassium was chosen according to our laboratory’s calibration. Serum potassium concentrations were measured daily from baseline (<24 h after admission) until discharge in all patients. For statistical analysis, the baseline value was computed. In order to gain insight into further analyses, we also considered patients with G4-G5 stage CKD, including 75 in group A and 46 in group B. Lastly, we considered patients with or without arterial hypertension.

The following variables were considered: age; serum potassium at admission serum creatinine at admission; eGFR (according to the CKD-EPI—Chronic Kidney Disease Epidemiology Collaboration formula [20]); therapy at admission (angiotensin-converting enzyme inhibitors (ACEIs), angiotensin II receptor blockers (ARBs), renin–angiotensin–aldosterone system inhibitors (RAASIs) (ACEIs and ARBs), loop diuretics, thiazides and potassium-sparing agents); comorbidities (arterial hypertension, diabetes mellitus and tumors); hemoglobin; and sum of hospitalization days (number of days on which the patients was hospitalized; we added up the days of hospitalization for all admissions for each patient).

### 2.3. Statistical Analysis

Categorical variables were expressed as frequencies and percentages; continuous variables were reported as medians and interquartile ranges because they were not normally distributed (Kolmogorov–Smirnov test for normal distribution). Only hemoglobin values were normally distributed, and they were reported as means ± standard deviation (SD). The differences in continuous variables were analyzed using the Mann–Whitney test, and the differences in prevalence for categorical variables were assessed with the Pearson’s chi-squared test. Spearman’s rank correlation test was used to find correlations between variables. Univariate and multivariable linear regression analyses were used to assess the independent effects of covariates on the sum of hospitalization days. Multivariate analyses were performed using all covariates significantly related with our outcome at univariate analysis. Estimated coefficients with 95% confidence intervals (CI) and related *p* values were reported. *p* values < 0.05 for two-sided tests were considered statistically significant. Statistical analysis was performed using SPSS software (version 17.0; IBM Corporation, Armonk, NY, USA).

Since the size of the sample to a power of 0.80 was not calculated, we cannot say that a non-significant result was equivalent to an actual absence of differences in inferential statistics.

## 3. Results

Table 1 summarizes the baseline characteristics of the 270 patients divided into group A (180 patients with potassium level ≤ 5.1 mEq/L) and group B (90 patients with potassium level > 5.1 mEq/L) including age, creatinine, eGFR, hemoglobin, sum of hospitalization days, ACEIs, ARBs, RAASIs considered globally, loop diuretics, thiazides, arterial hypertension, diabetes mellitus and tumors. Values are expressed as percentages, medians with interquartile range or means ± SD.

**Figure 1:**
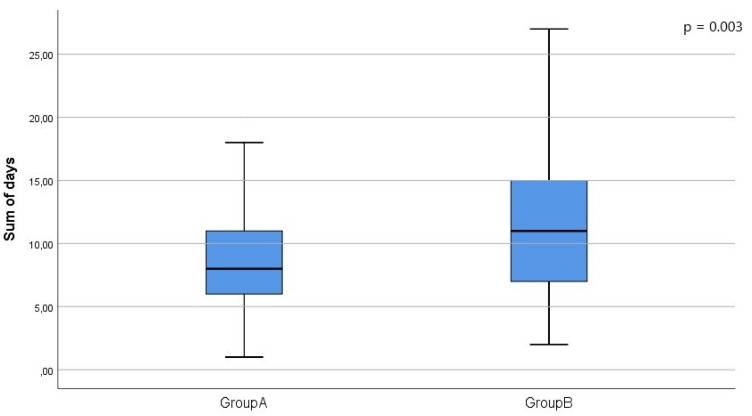
Boxplots showing the median and interquartile ranges (IQR) for sum of hospitalization days. The whiskers incorporate data that are 1.5 × IQR.

We observed significant differences between groups A and B for serum creatinine, eGFR, hemoglobin, sum of hospitalization days and use of ARBs, as shown in Table 1. We then considered patients with stage G4-G5 CKD: 84 in group A and 59 in group B. The two groups differed significantly only for sum of hospitalization days (9 (7–15) vs. 10 (9–17), *p* = 0.033) (Figure 1).

Table 2 shows correlations between age, creatinine, eGFR, hemoglobin, potassium and sum of hospitalization days in the entire sample, where the sum of hospitalization days was correlated with all variables evaluated. We may speculate that the length of hospitalization in these patients has multifactorial influence and that eGFR and hemoglobin are the most influencing factors.

Table 3 shows the results of univariate regression analyses in the entire sample of 270 patients considering the sum of hospitalization days as the dependent variable. Univariate analysis revealed that age and hyperkalemia were risk factors, whereas eGFR and hemoglobin were protective factors. Multivariable analysis confirmed that eGFR was an independent protective factor and that potassium level was an independent risk factor.

We then considered patients with eGFR between 6 and 30 mL/min/1.73 m^2^, excluding dialysis patients, and subdivided them according to potassium values into group A (K ≤ 5.1 mEq/L, *n* = 75 patients) and group B (K > 5.1 mEq/L, *n* = 46 patients). The baseline characteristics of the subsample with eGFR of 6–30 mL/min/1.73 m^2^ are presented in Table 4. Group A and group B differed significantly for days of hospitalization.

In univariate regression analysis in the subsample of patients with eGFR 6–30 mL/min/1.73 m^2^ (excluding dialysis patients), considering the sum of hospitalization days as the dependent variable, potassium was the only independent risk factor (β = 2.93, 95% CI, 0.077–5.794, *p* = 0.044); no associations were observed for other variables

**Figure 2:**
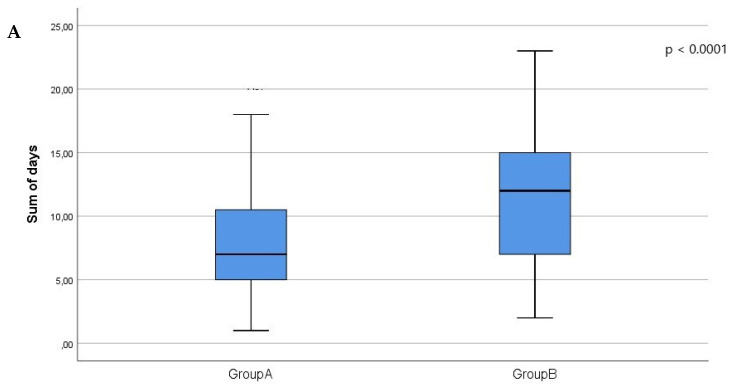

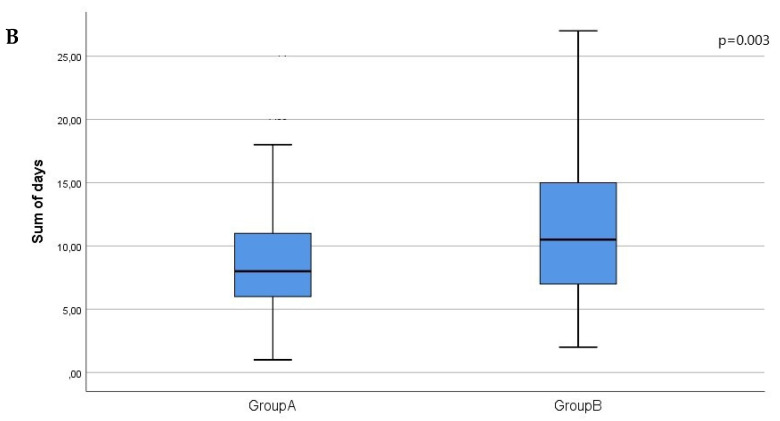
Considering those without arterial hypertension (**A**) and those with arterial hypertension (**B**), the figures show the sum of hospitalization days in group A (serum potassium ≤ 5.1 mEq/L) and group B (serum potassium > 5.1 mEq/L).

We also stratified patients in the entire study population according to the presence (AH group) or absence of arterial hypertension (non-AH) group: 80.3% of patients had arterial hypertension and 19.3% of patients showed no arterial hypertension. In group A, 77.2% of patients were in the AH group, and 22.8% were in the non-AH group; in group B, 86.7% of patients were in the AH group, and 13.3% were in the non-AH group. A significant difference in sum of hospitalization days was found between the two groups in the patients with arterial hypertension (group A, 8 [6,7,8,9,10,11] vs. group B, 11 [7,8,9,10,11,12,13,14,15]; *p* < 0.0001), as well as those without arterial hypertension (group A, 7 [5,6,7,8,9,10,11] vs. group B, 12 [7,8,9,10,11,12,13,14,15]; *p* = 0.003) (Table 5; Figure 2A,B).

## 4. Discussion

Hyperkalemia is a potential complication of the use of RAASIs. In a study involving 1589 hospitalized patients with acute HF, Beusekamp et al. [21] investigated the relationship between changes in dosage of RAASIs and the incidence of hyperkalemia. Patients with HF who developed hyperkalemia were under mineralocorticoid antagonist therapy before hospitalization and were more likely to have scaled back during hospitalization. The onset of hyperkalemia was associated with underdosing of mineralocorticoid antagonists, although patients who maintained or even increased the doses of these drugs (and/or ACEIs/ARBs) during hospitalization for acute HF had better 180-day survival. Although serum potassium concentrations increased in patients hospitalized for acute HF, hyperkalemia was not associated with adverse outcomes. Thus, in patients with HF, the possible onset of hyperkalemia can result in administration of doses of RAASIs that are too low [21].

In this observational study, we observed hyperkalemia in one-third of 270 patients admitted to the Unit of Nephrology and Dialysis of the University Hospital of Messina from 1 April 2018 to 31 December 2018 (T1). Comparing patients from group A (non-hyperkalemic, potassium level ≤ 5.1 mEq/L) with patients from group B (hyperkalemic group, potassium level > 5.1 mEq/L), hyperkalemic patients had a higher serum creatinine level, lower eGFR, slightly lower hemoglobin and greater sum of hospitalization days (Table 1).

Khanagavi and colleagues [22] investigated predictors of mortality in patients hospitalized with hyperkalemia (15,608 hospitalizations), excluding patients with end-stage renal disease on dialysis. CKD, acute kidney injury, metabolic acidosis, diabetes, hypertension, coronary artery disease and HF were frequent in hyperkalemic patients. Highest potassium level, tissue necrosis, metabolic acidosis, nonsteroidal anti-inflammatory drugs and acute kidney injury independently influenced the duration of hyperkalemia duration. Moreover, potassium supplementation, metabolic acidosis, treatment with calcium gluconate, acute kidney injury, tissue necrosis and long duration of hyperkalemia independently predicted in-hospital mortality.

If hyperkalemia is a risk factor for mortality, little is known about the impact of changes in potassium levels on the duration of hospitalization. In our study, comparing non-hyperkalemic patients (group A) with hyperkalemic patients (group B), hyperkalemic patients had higher sums of hospitalization days (Table 1).

In a cross-sectional study lasting for 4 months, Sarafidis et al. [23] analyzed the prevalence of hyperkalemia and related risk factors in 238 patients with different stages of CKD. Using univariate comparisons, the authors found that hyperkalemic patients had significantly higher levels of urea, lower eGFR and lower serum bicarbonate levels. Moreover, patients used sodium bicarbonate more often and received recommendations to reduce dietary intake of potassium. The use of ACEIs/ARBs was not associated with hyperkalemia. Low eGFR was the most important factor associated with hyperkalemia, and the frequency of hyperkalemia in patients with CKD pre-dialysis was high [23].

In our study, we found that hyperkalemia was associated with more severe CKD. Moreover, considering patients with G4-G5 stage CKD, we observed that hyperkalemic patients (group B) had higher sum of hospitalization days than non-hyperkalemic patients (group A) (Figure 1). These patients did not differ with regard to comorbidities and renal function; therefore, this finding strengthens the hypothesis of the impact of hyperkalemia on hospitalization.

In consideration of the beneficial effects of RAASIs in HF and CKD, dietary and therapeutic strategies are needed to allow correct control of hyperkalemia in patients who take advantage of these drugs. Our study did not show significant differences in the use of RAASIs before admission between hyperkalemic and non-hyperkalemic patients probably due to the size of simple, but we can suppose that the use of these drugs can be possible with correct nutrition education and correct use of potassium binders.

Factors reducing renal function also increase the risk of hyperkalemia. Loutradis et al. [24] studied the prevalence of hyperkalemia in diabetic and non-diabetic patients with CKD. The authors found that diabetes mellitus increased the prevalence of hyperkalemia only in patients with moderately impaired renal function (CKD stage III), whereas CKD stage G4 and ACEIs were the main determinants of hyperkalemia [24]. In our study, we did not find differences in the prevalence of diabetes between hyperkalemic and non-hyperkalemic patients.

Making a correlation between variables, we observed that potassium levels were inversely correlated to hemoglobin and eGFR. The sum of hospitalization days was negative correlation to eGFR and hemoglobin and directly correlated to age and creatinine level (Table 2). We then used univariate and multivariable regression analyses in the entire sample of 270 patients to identify factors considering the sum of hospitalization days as the dependent variable (Table 3). Univariate analysis revealed that diabetes and potassium level are independent risk factors, and eGFR and hemoglobin are independent protective factors (Table 3). Multivariable analysis confirmed eGFR as an independent protective factor and potassium level as an independent risk factor (Table 3).

In the entire sample, univariate regression analysis showed an association between diabetes and sum of hospitalization days, hemoglobin and sum of hospitalization days, potassium level and sum of hospitalization days and eGFR and sum of hospitalization days. Multivariable regression analysis confirmed the independent role of eGFR and hyperkalemia. Moreover, in the subsample of patients with eGFR of 6–30 mL/min/1.73 m^2^, we used univariate regression analysis by considering sum of hospitalization days as the dependent variable, and we observed that hyperkalemia was the only independent risk factor; there was no association with diabetes, eGFR and hemoglobin. These data confirm our hypothesis that the impact of hyperkalemia on hospitalization is independent of the confounders considered.

In order to strengthen the analysis, we considered patients with and without arterial hypertension. Stratified analysis confirmed an increase in the sum of hospitalization days in patients in group B with and without arterial hypertension (Table 5). Overall, our data support the hypothesis that hyperkalemia has a relevant impact on hospitalization.

The available sample size allowed evidence for statistical significance of the observed clinically relevant differences.

A limitation of our study is that we cannot establish if high serum potassium levels per se are responsible for the observed increase in length of hospital stay and risk for hospitalization, or if they are rather an epiphenomenon of more severe clinical conditions. High potassium-related arrhythmias can be responsible for worsening clinical conditions and increases in hospitalization. Another potential explanation could be linked to the evidence that increased ammoniagenesis may enhance kidney growth and injury, as reported in some models including the remnant kidney, experimental diabetic nephropathy, high protein feeding, hypokalemic nephropathy and dietary deficiency of antioxidants [25]. Hyperkalemia reduces ammoniagenesis, discouraging this compensatory mechanism. Although ammonia induces complement cascade activation, ammoniagenesis is important to maintain renal acid excretion end nephron survival [26]. Wesson DE et al. reported as urine ammonia is correlated with urine TGFβ1 and as chronic metabolic acidosis increases inflammation and fibrosis [27]. The review highlighted that, in animal models, acid exposure increased tubular necrosis and increased oxidative stress, and treating metabolic acidosis reduced kidney damage in terms of reduced production of cell nuclear antigen and N-acetyl-β-D-glucosaminidase. It cannot be excluded that unknown chronic consequences of hyperkalemia play a role in the mismanagement of this condition.

A further limitation of this study design is that it does not allow us to determine whether correction of hyperkalemia may reduce the number and duration of hospitalizations.

## 5. Conclusions

In our study, despite the absence of differences in baseline values between non-hyperkalemic and hyperkalemic patients, we observed a significantly increased length of hospital stay. These data are more evident in patients with CKD stage G4 and G5, when potassium levels seem to be one of the main risk factors. Moreover, the frequency of hyperkalemia had a tendency with the use of RAASIs, refuting the tendency to contraindicate these drugs in cases of CKD but supposing a contemporary use of these with a strong follow up of hyperkalemia and pharmacological treatment of these adverse events. These results allow us to hypothesize that good pharmacological control of side effects allows us to use this class of drugs in patients with impaired renal function.

Further studies are needed to investigate if our findings are influenced by other more severe clinical conditions linked to reduced ammoniagenesis or inadequate therapeutic management of hyperkalemia at all stages of severity.

## Figures and Tables

**Table 1:** Baseline characteristics of patients in group A (180 patients, serum potassium ≤ 5.1 mEq/L) and group B (90 patients, serum potassium > 5.1 mEq/L).

	Group A (*n* = 180)	Group B (*n* = 90)	*p*
Age, years	76 (64–81)	76 (66–82)	0.330
Creatinine, mg/dL	1.7 (1.1–3.2)	2.6 (1.7–4.6)	**<0.001**
eGFR, mL/min/1.73 m^2^	34.89 (16.24–57.98)	19.8 (10.50–32.50)	**<0.001**
Hemoglobin, g/dL	11.64 ± 2.20	10.97 ± 2.19	**0.04**
Sum of hospitalization days	8 (6–10)	11 (7–15)	**<0.001**
ACEIs, *n* (%)	36 (20.1)	16 (17.8)	0.56
ARBs, *n* (%)	72 (40.2)	48 (53.3)	**0.041**
Loop diuretics, *n* (%)	77/9 (43.0)	46 (51.1)	0.209
Potassium sparing agents, *n* (%)	19 (10.6)	7 (7.8)	0.428
RAASIs, *n* (%)	105 (58.7)	62 (68.9)	0.103
Thiazides, *n* (%)	22 (12.3)	6 (6.7)	0.162
Arterial hypertension, *n* (%)	139 (77.7)	78 (35.9)	0.077
Diabetes mellitus, *n* (%)	70 (40.0)	33 (38.4)	0.800
Heart failure, *n* (%)	3 (1.7)	4 (4,4)	0.227
Tumors, *n* (%)	15 (8.4)	9 (10.0)	0.88

Data are expressed as the median (interquartile range) for non-Gaussian distribution, mean ± SD for Gaussian distribution or absolute number and percentage for dummy variables. *p* values were obtained by the Mann–Whitney test for continuous variables and by chi-squared test for categorical variables. Bold values indicated *p* < 0.05. ACEIs, angiotensin-converting enzyme inhibitors; ARBs, angiotensin II receptor blockers; eGFR, estimated glomerular filtration rate; RAASIs, renin-angiotensin-aldosterone system inhibitors.

**Table 2:** Correlations between variables in the entire sample.

		Age, Years	Creatinine, mg/dL	eGFR, mL/min/1.73 m^2^	Hemoglobin, g/dL	Potassium, mEq/L	Sum of Hospitalization Days
Age, years	Rho						
	*p*						
Creatinine, mg/dL	Rho	0.088					
	*p*	0.151					
eGFR, mL/min/1.73 m^2^	Rho	−0.199	−0.977				
	*p*	0.001	0.000				
Hemoglobin, g/dL	Rho	−0.261	−0.360	0.390			
	*p*	0.000	0.000	0.000			
Potassium, mEq/L	Rho	0.072	0.290	−0.286	−0.147		
	*p*	0.239	0.000	0.000	0.017		
Sum of hospitalization days	Rho	0.211	0.317	−0.337	−0.272	0.175	
	*p*	0.001	0.000	0.000	0.000	0.004	

eGFR, estimated glomerular filtration rate.

**Table 3:** Univariate and multivariable regression analyses in the entire sample of patients (*n* = 270) considering the sum of hospitalization days as the dependent variable.

Variables	Univariate Analysis			Multivariable Analysis		
	Beta	95% CI	*p*	Beta	95% CI	*p*
Age (years)	0.066	0.013/0.119	0.016	0.005	−0.052 /0.061	0.869
eGFR (mL/min/1.73 m^2^)	−0.069	−0.095/−0.043	<0.001	−0.052	−0.083/−0.021	0.001
Hemoglobin (g/dL)	−0.681	−1.041/−0.320	<0.001	−0.360	−0.739/0.02	0.065
Hyperkalemia (no—yes)	3.089	1.416/4.762	<0.001	1.917	0.208/3.625	0.028

eGFR, estimated glomerular filtration rate.

**Table 4:** Baseline characteristics of the two study groups in the subsample of patients with eGFR of 6–30 mL/min/1.73 m^2^, excluding dialysis patients (group A with serum potassium ≤ 5.1 mEq/L; group B with serum potassium > 5.1 mEq/L).

	Group A (*n* = 75)	Group B (*n* = 46)	*p*-Value
Age, years	77 (72–82)	75 (67–84)	0.533
Creatinine, mg/dL	3.3 (2.5–4.00)	3.45 (2.5–4.5)	0.677
eGFR, mL/min/1.73 m^2^	15.45 (10.7–23.27)	15.41 (10965–23.27)	0.548
Hemoglobin, g/dL	10.69 ± 1.98	10.88 ± 1.96	0.404
Sum of hospitalization days	9 (7–15)	10 (9–17)	0.033
ACEIs, *n*/%	12 (16.0)	10 (21.7)	0.427
ARBs, *n*/%	25 (33.3)	20 (43.5)	0.262
Loop diuretics, *n*/%	42 (56.0)	23 (50.0)	0.521
Potassium sparing agents, *n*/%	10 (13.3)	5 (10.9)	0.690
RAASIs, *n*/%	36 (48.0)	30 (65.2)	0.065
Thiazides, *n*/%	1 (1.3)	3 (6.7)	0.115
Arterial hypertension, *n*/%	61 (81.3)	42 (91.3)	0.135
Diabetes mellitus, *n*/%	37 (50.0)	15 (34.1)	0.092
Heart failure, *n* (%)	1 (1.2)	4 (8.7)	0.162
Tumors, *n*/%	9 (12.0)	3 (6.5)	0.371

Data are expressed as the median (interquartile range) for non-Gaussian distribution, mean ± SD for Gaussian distribution or absolute number and percentage for dummy variables. *p* values were obtained by Mann–Whitney test for continuous variables and by chi-squared test for categorical variables. ACEIs, angiotensin-converting enzyme inhibitors; ARBs, angiotensin II receptor blockers; eGFR, estimated glomerular filtration rate; RAASIs, renin-angiotensin-aldosterone system inhibitors.

**Table 5:** Differences between group A (serum potassium ≤ 5.1 mEq/L) and group B (serum potassium > 5.1 mEq/L) with regard to sum of hospitalization days, stratifying patients according to the presence or absence of arterial hypertension.

	Sum of Hospitalization Days		
	Group A	Group B	*p*
No arterial hypertension (*n* = 52)	7 (5–11)	12 (7–15)	0.006
Arterial hypertension (*n* = 217)	8 (6–11)	11 (7–15)	0.003

Data are expressed as medians (interquartile range) for non-Gaussian distribution. *p* values were obtained by Mann–Whitney test.

## Data Availability

The data that support the findings of this study are available from the corresponding author upon reasonable request.
